# Mechanisms, Functions, Research Methods and Applications of Starch–Polyphenol Complexes in the Synergistic Regulation of Physiological Parameters

**DOI:** 10.3390/foods14183219

**Published:** 2025-09-17

**Authors:** Zhehao Hu, Yanyan Xu, Yuanqian Xiong, Ganhui Huang

**Affiliations:** State Key Laboratory of Food Science and Resources, Nanchang University, Nanchang 330047, China; shanyewudengsuozhang@gmail.com (Z.H.); beishangguobaorou22@gmail.com (Y.X.);

**Keywords:** starch–polyphenol complexes, empty V-type inclusion complexes, slowly digestible starch (SDS), resistant starch (RS), low glycemic index (GI)

## Abstract

Metabolic illnesses such as obesity, type 2 diabetes and hyperuricemia are becoming more common, driving intensified research into nutritional interventions through targeted dietary modifications as a primary preventive strategy. The apparent fluctuation in blood glucose value is modulated by the digestive behavior of starch. Moreover, polyphenols—historically considered to be anti-nutrients due to their inhibition of digestive enzymes and sometimes astringent taste—can be used to significantly improve the functional properties of starch. This can be achieved primarily through α-amylase inhibition and the modulation of other enzyme activities, alongside the antioxidant and anti-inflammatory effects of polyphenols. Depending on their fine structure, starches are digested at different rates: rapidly digestible starch (RDS) spikes blood glucose; slowly digestible starch (SDS) smooths postprandial blood glucose peaks; resistant starch (RS) feeds gut microbes. The fine structure of starches, such as straight-chain starches, can form complexes with polyphenols through their ‘empty V-type’ structures under controlled processing conditions. Fourier-transform infrared spectroscopy (FTIR), X-ray diffraction (XRD) and in vitro digestion modeling analyses have revealed that the formation of starch–polyphenol complexes primarily occurs due to certain interactions (hydrophobic interactions and hydrogen bonding) which lead to stabilized structures, including V-type encapsulation; this significantly increases the content of RSs and slows down enzymatic digestion rates. These complexes lower the GI values of foods via molecular barrier effects, while synergistically boosting antioxidant and anti-inflammatory activities; their anti-digestive capabilities were found to be superior even to those of ordinary starch–lipid compounds. However, limitations persist in the research and application of starch–polyphenol complexes: human bioavailability validation; incomplete mechanistic understanding of multicomponent interactions; industrial scalability challenges due to polyphenol instability.

## 1. Introduction

Photosynthesis, which converts light energy to chemical energy and is stored primarily as starch by plants, is a key mechanism by which plants adapt to their surroundings and maintain life. Starch is the principal energy storage molecule in plants. The digestibility rate of starch has a direct impact on blood glucose swings and energy supply stability [1,2]. In current dietary patterns, excessive consumption of rapidly digestible starch (RDS) has been demonstrated to be directly associated with the development of metabolic disorders such as obesity and type 2 diabetes [3]. Consumption of slowly digestible starch (SDS) and resistant starch (RS) influences the peaks and valleys of postprandial blood glucose levels, whereas the latter provides a carbon source for intestinal microflora to produce short-chain fatty acids (SCFAs). These SCFAs have been shown to use intestinal receptor cells to secrete hormones such as glucagon-like peptide-1 (GLP-1) and leptin, which regulate physiological parameters. Long-term consumption can significantly alter the structure of the intestinal flora as well as the metabolic profiles of SCFAs, improving a host’s blood glucose tolerance and insulin resistance. However, the magnitude of this regulatory effect may diminish over prolonged periods of continuous intake, highlighting the necessity of a synergistic intervention strategy [4]. Therefore, the fine structure of raw starch, the effect of processing on starch structure and the kinetic characterization of the digestive process have become important research directions in the support of formulating dietary guidance that can be used to regulate blood glucose and improve physiological indices.

Plants synthesize polyphenols as evolved defenses against biological and chemical injuries such as insect and animal feeding and the occurrence of free radicals and oxidation during growth. This process integrates multiple functions such as defense, structural support, signaling and ecological interactions. Polyphenols, as secondary metabolites in plants, are involved in scavenging the reactive oxygen species produced during photosynthesis and the regulation of cellular antioxidant defense; they also lead to enhanced ultraviolet light and biotic stress resistance through the activation of pathogen defense signaling pathways [5]. In addition to the dominating chemical features of antioxidants, the binding reaction of plant polyphenols with proteins—which are the material basis of all living phenomena and have metabolic regulation properties—is their most important chemical feature; during the last century, researchers had previously perceived polyphenols negatively due to their inhibition of digestive enzymes and undesirable taste. With advancements in the economy and in our theoretical understanding, food-borne polyphenols have increasingly become a research hotspot in the fields of nutrition and functional foods. For instance, they have been found to successfully lower the postprandial glycemic response by suppressing the activity of digestive enzymes involved in the breakdown of carbohydrates, such as α-amylase [6]. Although α-glucosidase inhibitors like acarbose can reduce postprandial blood glucose peaks, they frequently have a restricted safety window due to their gastrointestinal side effects and poor tolerance [7]. In order to achieve gentle and sustained blood glucose regulation with a natural slow-release effect and a multicomponent synergistic effect, as well as antioxidant, anti-inflammatory and other comprehensive health benefits, polyphenol-based dietary regimens must have active ingredients incorporated into their daily dietary matrices. As a result, they have a wider safety window to better meet the requirement of helping in the long-term management of metabolic diseases [8].

Furthermore, polyphenols form compounds with starch molecules by hydrophobic hydrogen bonding or electrostatic interactions, changing the crystalline structure and thermal stability of starch and slowing enzymatic activity, thus dramatically increasing the fraction of RSs [9,10]. Therefore, more research should be focused on the relationship between starches, polyphenols and physiological markers.

The functional properties of starch–polyphenol synergisms, such as postponing digestion [3], lowering GI [11], enhancing antioxidant and anti-inflammatory activities [10] and scavenging free radicals [10], have been validated by a number of fundamental studies. However, there is still a significant lack of in vivo assessments of the bioavailability and intestinal bacterial flora regulation of starch–polyphenol synergisms, and there is a lack of validation of their long-term health effects; therefore, we have an unclear understanding of the association between function and safety in practical applications of starch–polyphenol synergisms. It is necessary to conduct molecular analyses of the following aspects: their mechanisms of action; the evolution of crystalline structures during complex formation; the intrinsic correlation between the enzyme inhibition effect and the change in the value of RSs; the stereospecific binding mode of polyphenol molecules and (straight-/branched-chain) starches. The lack of systematic comparative studies covering multiple polyphenol classes and different starch fractions limits our understanding of their complex formation. On the theoretical level, the current results are primarily focused on validating the interaction between a single type of polyphenol (such as flavonoids or phenolic acids) and starches. Although the structure–function synergistic mechanism of these complexes has not yet been fully understood on the practical level, their potential in three main areas—intestinal microecology optimization, postprandial blood glucose regulation and food shelf-life extension—has been tentatively identified. However, more research is required if we are to fully understand the precise pathways, dosage–effect relationships and important technical issues, like processing adaptations, of these complexes.

Building on these discoveries, this narrative review methodically summarizes the mechanisms of interaction between polyphenols and starch, the physiological effects of their complexes, their physicochemical characteristics, their characterization techniques and their potential uses in food science. With an emphasis on determining how polyphenols alter the physicochemical characteristics of starches, this review analyzes the potential of and difficulties encountered in employing contemporary analytical methods to decipher structure–function links in these complexes, with the aim of supporting the development of new health-conscious foods. The present review aims to show how advanced characterization technologies can be used to reveal structure–property correlations, identify novel applications in low-GI foods and smart packaging and illuminate industrial implementation barriers. This review also aims to clarify the molecular mechanisms by which polyphenols regulate starch digestibility and functionality through intermolecular interactions. Through the integration of scientific knowledge with real-world applications, this review presents new insights into starch–polyphenol complexes and offers a theoretical and technical framework for creating food systems that are both functionally improved and nutritionally balanced.

## 2. Structures of Starches and Polyphenols and Their Physiological Roles

### 2.1. Fine Structure of Starch and Its Origin

Starch plays different physiological roles due to the diversity of its fine structures, such as the ratio of straight chains to branched chains, molecular weight, chain length distribution, branching degree and crystalline form. Straight-chain starches are distinguished by their longer linear chains (DP > 1000) and double-helix shape; they are characterized by their high resistance to digestive enzymes, resulting in a large concentration of RSs. Branched starches are primarily composed of short–medium, highly branched chains (DP 10–60) which expand the spatial structure after pasting and reveal more enzyme action sites. As a result, postprandial blood glucose levels rise quickly due to fast hydrolysis. In native starch granules, crystallinity predominantly reflects the organized packing of amylopectin, while linear amylose is largely amorphous; however, following gelatinization and subsequent processing (e.g., retrogradation or inclusion-complex formation), amylose can reassociate into V-type or retrograded crystalline domains that substantially contribute to the crystallinity, thermal behaviour and RS content of processed starches. Different crystalline kinds (A, B and C) have varying degrees of accessibility to digestive enzymes: A-type crystals have been shown to be the most vulnerable to enzymatic breakdown, while B-type crystals have been shown to be the most resistant to digestion. To create low-GI or slow-release energy starch products with various digestive qualities, researchers have carefully regulated the proportion of straight-chains to branched-chains, and have regulated the molecular weight distribution and crystalline types of starches from maize, wheat, rice and other grains—as well as potato, cassava and other yams—using physical (heat treatment or shearing), chemical (cross-linking or esterification) and enzymatic (branched enzyme or debranched enzyme) methods [1,2]. Starch is an essential source of carbohydrates for both humans and animals, which can be found in beans, potatoes, corn, wheat, rice and other plants [1,2]. Maize is one of the primary sources of industrial starch worldwide. Maize starch is widely used in the food industry and as a biodegradable material. [1]. In South Asia, rice starch provides a substantial amount of dietary calories [2]. Furthermore, in low-income nations, yams, like potatoes and cassava, provide a significant source of energy [3].

Moreover, the different botanical origins of starch engender distinct physicochemical signatures that reflect each plant’s unique biosynthetic pathways and storage tissue architectures. For instance, pea starch, recovered as a byproduct of pea protein processing, comprises over 50% of a seed’s dry weight and forms relatively small, spherical granules, owing to the legume-specific expression of branching and debranching enzymes in its amyloplasts [12]. Conversely, cassava starch is synthesized in and stored by storage root plastids, leading to large, irregular granules that promote rapid hydration and swelling, which is an adaptation seen in subterranean carbohydrate reserves [13]. These interspecies disparities can be traced back to the differential activities of starch synthases, branching enzymes and debranching hydrolases, as well as the organizational framework of their respective amyloplasts; these observations underscore the importance of source selection when tailoring extraction methods and functional applications in this field [14].

### 2.2. Starch Digestion and Postprandial Blood Glucose

Starch enters the body and is eventually digested and turned to blood glucose, providing energy. Starches are digested at different speeds depending on their structural types. RDS causes a sharp rise in blood glucose after meals because it is frequently completely digested in 20 min. Long-term consumption of foods high in RDS is strongly linked to metabolic disorders such as type 2 diabetes and obesity [3]. SDS takes up to 120 min to fully hydrolyze, providing long-lasting energy and helping to stabilize blood glucose levels [7]. RS is not easily digested by the small intestine and is fermented by gut microorganisms after entering the colon to produce short-chain fatty acids (SCFAs), which help to improve intestinal barrier function and reduce inflammatory risk [1]. Recent work has further shown that a fifth category, RS V, can be generated when linear amylose chains form V-type inclusion complexes with hydrophobic guests such as polyphenols; these complexes retain the V-helix signature, yet they resist in vitro enzymatic breakdown and produce enhanced prebiotic effects during fermentation, thereby extending the mechanistic framework of starch resistance beyond purely physical or enzymatic factors [15]. Furthermore, the GI of food varies according to how different types of starch affect the rate of digestion. Low-GI foods often help prevent metabolic disorders like obesity and type 2 diabetes by reducing the dramatic fluctuations that can occur in postprandial blood glucose due to the slower release of blood glucose [1]. In order to improve the health benefits of starch, modern technology reduces the rate of starch digestion through physical or chemical modifications (e.g., hot–wet treatment and debranched starch recrystallization) or further inhibits its excessively rapid digestion by combining it with polyphenols to form complexes [3]. Moreover, when these modification methods are combined with the in situ formation of RS V via guest molecule inclusion, the resulting starches display even higher resistant starch content and greater suppression of postprandial glycemic responses [15].

The readiness by which starch can be digested is directly influenced by the number of ‘contact sites’ the starch has. Besides its function as an energy source, the main physiological significance of starch is found in its rate of digestion and resistance to enzymatic breakdown in the upper gastrointestinal tract. However, the percentage of branched-chain starches in cereals and other foods is rising due to peoples’ desire for soft texture, easy digestion and easy absorption. As a result, the synergistic effect of starch and polyphenols in food on physiological indicators becomes more significant when the ‘food diversity’ principle and sensible cooking techniques are applied. The way in which starch and polyphenols in food work together to regulate physiological parameters thus becomes clearer.

### 2.3. Source and Bioavailability of Polyphenols

Hydroxyl is a common polar group, and the presence of hydroxyl makes the molecule more polar as a whole, exhibiting its physiological value. Polyphenols—which have multiple phenolic hydroxyl (-OH) structural units—can be classified into several categories based on their chemical structure: flavonoids, polyphenolic acids, ellagic acids, anthocyanins, etc. Notable common polyphenol compounds include catechins (EGCG) in tea, resveratrol in grapes and hesperidin in citrus fruits [6,16]. Polyphenols have been found in a variety of foods, particularly fruits, vegetables, tea and whole grains. Citrus fruits are rich in hesperidin [6]; blueberries are rich in anthocyanins [17]; catechins (EGCG) in green tea are polyphenol components with considerable health benefits [16]. In addition, agricultural byproducts such as grape pomace, olive pomace and walnut shells are rich sources of polyphenols [18]. Some specialty plants such as butterfly pea and yerba mate are also rich in polyphenols, which have antidiabetic and anti-obesity health benefits [16]. Plant foods that are high in polyphenols are principal sources of polyphenols for the human body; these include fruits (citrus, blueberries, etc.), vegetables (spinach), whole grains (black rice, quinoa, etc.) and teas (green tea, black tea, etc.). The processes of digestion and absorption are influenced by a number of factors, including the alkaline environments made by the small intestine, pancreatic enzymes and gastric acid. Certain polyphenols, like hesperidin, are released more readily when gastric acid is present, whereas pepsin may hinder the release of some polyphenols or even break down their structure [18]. The bioavailability of polyphenols in the small intestine was found to be enhanced by the alkaline environment and presence of pancreatic enzymes. Nevertheless, during digestion, some polyphenols may be oxidized or bound to other food ingredients, which would reduce their actions. Modern technologies frequently employ nanoencapsulation and enrichment encapsulation techniques to improve the stability and bioavailability of polyphenols, despite their differing principles [16]. Additionally, the lactic acid bacteria fermentation method was found to greatly increase the antioxidant activity of polyphenols. For instance, after lactic acid bacteria was used to ferment hawthorn pulp, the antioxidant activity of polyphenols increased and their bioavailability increased by 40% [19].

## 3. Interactions Between Starch and Polyphenols and Their Physiological Value

### 3.1. Interaction of Flavonoid Polyphenols with Starch

Flavonoid polyphenols, including quercetin (Qu) and rutin (RT)—with multiple phenolic hydroxyl groups (-OH) and hydrophobic aromatic ring structures (e.g., benzene and pyran rings) with strong antioxidant effects—often interact with starch molecules through hydrophobicity and hydrogen bonding, affecting the physicochemical properties of starch [20]. Notably, the binding specificity of flavonoids is governed by their structural subclasses. For instance, quercetin embeds deeply into the helical cavity of straight-chain starch via hydrophobic interactions, increasing RS content [9]. In contrast, catechins, such as EGCG, lack the C4-carbonyl and instead form multi-point hydrogen bonds with branched starch surfaces due to their higher hydroxyl density, resulting in lower RS but enhanced antioxidant stability [9,16]. Rutin’s glycosylation further shifts its binding toward electrostatic adsorption [21], demonstrating how subtle structural variations dictate functional outcomes.

**Figure 1:**
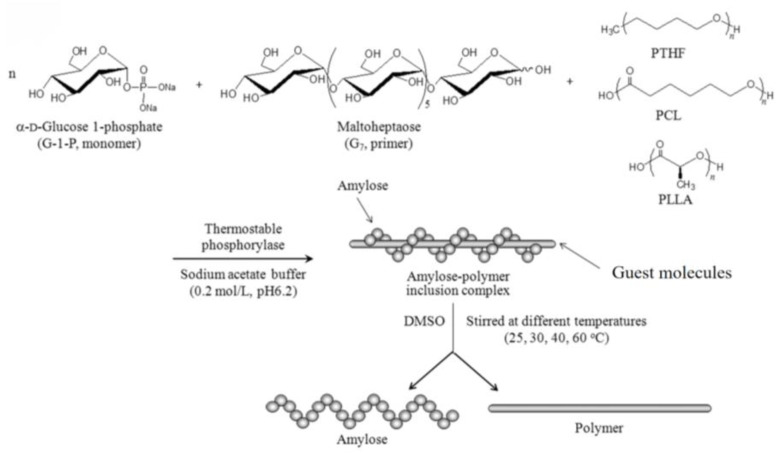
Molecular model of amylose V-type inclusion complex formation with hydrophobic guest molecules. Reprinted with permission from the authors of Ref. [26], 2017: Tomonari Tanaka, Atsushi Tsutsui, Kazuya Tanaka, Kazuya Yamamoto and Jun-ichi Kadokawa.

**Figure 2:**
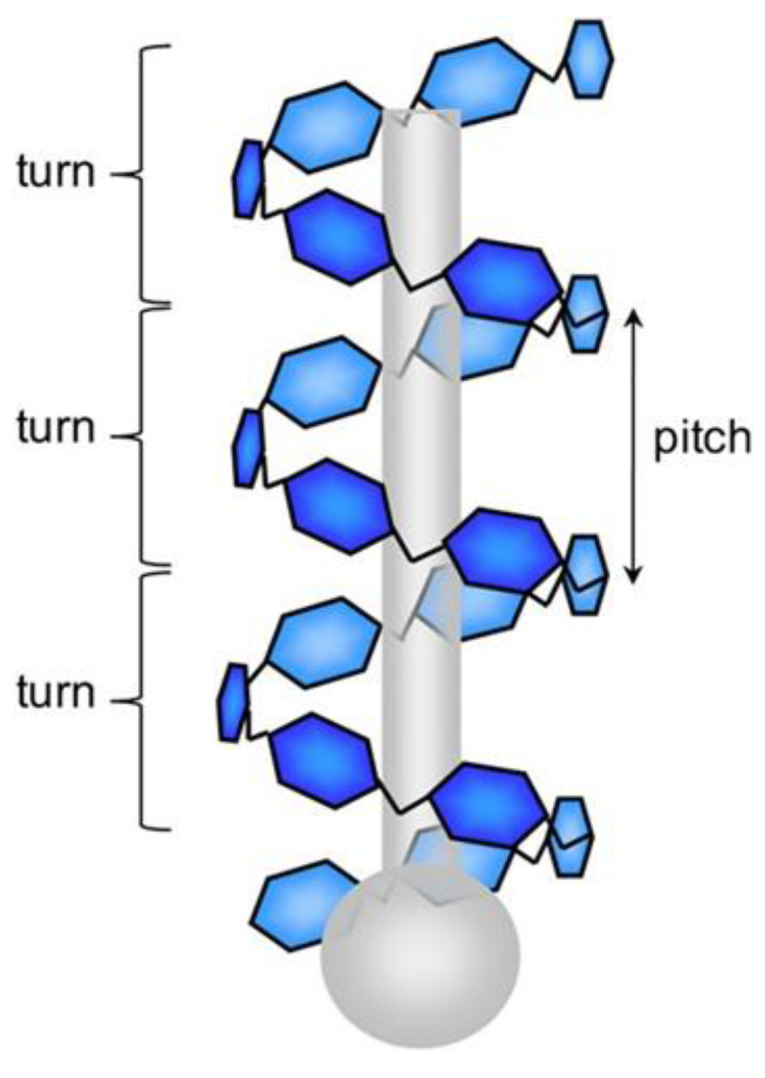
Molecular model of amylose V-type inclusion complex formation with hydrophobic guest molecules. Adapted with permission from the authors of Ref. [27], 2006: Lingyun Chen, Gabriel E. Remondetto and Muriel Subirade.

Miao’s team concluded that quercetin, through its hydrophobic structure and hydrogen bonding, binds to the helical cavity of straight-chain starch to form a V-shaped inclusion, which enhances the RS content of high-temperature straight-chain maize starch from 8.3% to 32.5% and retards the digestion rate by more than 40%. Additionally, the complex’s thermal stability is greatly improved (the decomposition temperature rises from 300 to 325 °C), making it appropriate for use in high-temperature environments. Finally, the complex’s antioxidant activity (a 1.8-fold increase in DPPH radical scavenging rate) makes it suitable for use in high-temperature environments as well. Chen et al. revealed the formation of a V-type inclusion complex, as shown in Figure 1. Rutin, as a negatively charged flavonoid, combines with the surface charge of branched starch to form surface adsorption complexes mainly through electrostatic interactions. Zhang et al. discovered that the phenolic hydroxyl group of rutin forms multiple hydrogen bonds with the blood glucose units at the branching points of branched starch. At the same time, its aromatic ring binds to the hydrophobic regions of the starch chain through π-π stacking, which not only increases the stability of the complexes but also significantly reduces the contact probability between α-amylase and starch molecules through the spatial site-blocking effect. This could increase the RS content of potato branched-chain starch from 5.1% to 18.7% and lower the enzymatic rate by 35%. It was found to be useful in the field of food preservation (e.g., inhibition of lipid oxidation) because of its antioxidant properties (2.2-fold increase in the scavenging rate of ABTS free radicals) [20]. In contrast to other complex types, type II single-helix inclusion complexes (e.g., lauric acid–starch complexes) embed linear molecules in the starch helical cavity through hydrophobic interactions, which elevate the RS content to only 15–20% (significantly lower than type-V inclusion complexes) and are less thermally stable (with a decomposition temperature of about 280 °C) [22]. Networked amorphous cross-linking structures, such as protocatechuic acid–starch hydrogen-bonded complexes, are created by multi-point hydrogen bonding to create a disordered network. Although the RS content is between 10% and 15%, the V-type system has stronger antioxidant and digestive resistance [23]. The mechanism of action and the benefits of the properties of V-type inclusion complexes have given theoretical support for precise nutritional interventions. When combined, these complexes are the best way to control the functional properties of starch to produce low-GI foods owing to the molecular-barrier mechanism, the largest increase in RS (up to three to four times that of the original starch), and the synergistic enhancement of thermal and antioxidant stability. Chen revealed in Figure 2 that the steric conformation prevents enzymes from contacting the starch chain [9,20]. It is worth noting that recent studies have further optimized the preparation process of V-type complexes by preforming starch helical cavities via the ‘empty V-type method’. According to Guo et al., starch helical cavities can be effectively embedded in lipophilic molecules like ascorbyl palmitate (AP) at 70 °C. The complex’s crystallinity reaches 17.68%, and its thermal decomposition temperature rises to 96 °C. When annealing and acid hydrolysis are used in tandem, the complex’s RS content can increase from 2% to 51% [24]. In contrast, the traditional dimethyl sulfoxide (DMSO) solubilization approach involves a high-temperature crystallization process accompanied by a lengthy heating period in a boiling water bath to dissolve starch. This procedure not only uses a lot of energy but also has the potential to destabilize heat-sensitive bioactive components (like polyphenols) [25]. The empty V approach’s low temperature and high efficiency can elude the high temperature restriction encountered with the traditional DMSO method; moreover, it can open up a new avenue for the industrial production of low-GI food products. This further emphasizes the dual benefits of V complexes in terms of structure design and function enhancement.

### 3.2. Interaction of Astragalus Polyphenols with Starch

**Figure 3:**
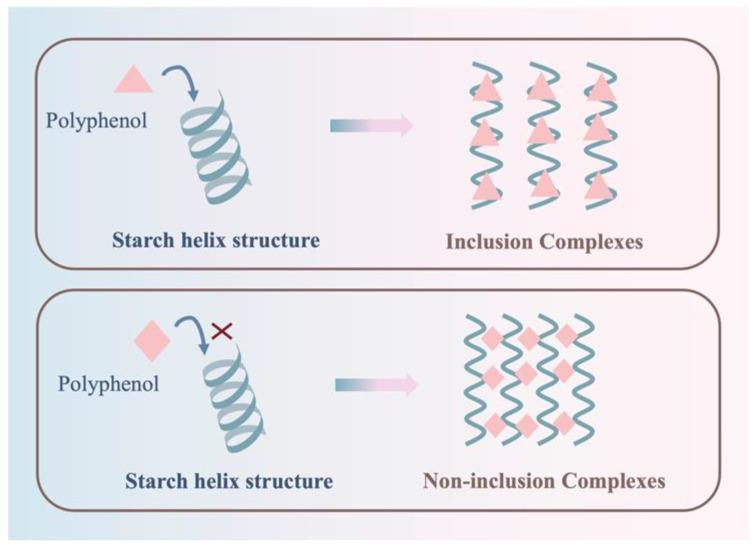
Comparison of linear (amylose-rich) starch forming a V-type inclusion and branched (amylopectin-rich) starch forming multilevel network cross-linking. Adapted with permission from the authors of Ref. [21], 2024: Yingying Wu, Yanan Liu, Yuanqiang Jia, Huijuan Zhang and Feiyue Ren.

Astragalus polyphenols such as resveratrol and its natural derivative piceatannol (3,4,3′,5-tetrahydroxystilbene) are all based on a stilbene matrix structure with two benzene rings linked by an ethylene bridge; these compounds are present in plants in very small amounts and are mainly found in the thin-walled cells of the xylem. Mengji Dong et al. [28] loaded piceatannol on corn starch aerogel using a supercritical fluid impregnation technique; this technique can be used to produce V-type encapsulation with hydrophobic single-helix cavities in the molecular chain of straight-chain starch. The phenolic hydroxyl lone pair of electrons on the piceatannol molecule effectively overlaps with the σ-anti-bonding orbitals of the hydroxyl hydrogens on the starch chain to form a strong hydrogen bond; the π–electron cloud of the aromatic ring of the piceatannol molecule produces weak π–σ interactions with the C-O σ orbitals of the helical cavities of straight-chained amylopectin, which microscopically enhances the thermal stability of the inclusion complexes and the resistance to enzymatic degradation through the σ–π synergistic effect. Resveratrol is capable of driving the assembly of V-type inclusion complexes with the help of its phenolic hydroxyl group. This overlaps with the σ–σ orbitals of the amylose hydroxyl group and the CH–π interactions of the aromatic ring’s π–electron bonds; here, the C–H bond in maize starch systems has different straight-chain contents, which can lead to the dissociation of the double-helix network and its reconstruction into a single-helix/semi-crystalline structure, thus reducing the in vitro digestion rate and increasing the resistant starch content [29]. Building on this, as illustrated in Figure 3, linear (amylose-rich) starch preferentially forms a V-type single-helix inclusion; in contrast, branched (amylopectin-rich) starch adopts multilevel network cross-linking or encapsulation. This distinction is aligned with the enhanced thermal stability and resistance to enzymatic degradation. Compared to piceatannol, the interaction of resveratrol with branched-chain starch is more complex. While aromatic ring π–π-electrons interact weakly with hydrophobic groups on the surface of branched starch at multiple levels of π–σ interactions, its phenolic hydroxyl group also forms multiple hydrogen bonding networks with hydrogen atoms in branched starch chains through σ* orbitals. This leads to the formation of a multistage V-type encapsulated or non-encapsulated complex that is stable and thermoresistant at the molecular level at branching points. This increases the RS concentration and thermostability of branched-chain starch, while simultaneously inhibiting α-glucosidase activity and reducing blood glucose release during digestion [30]. The mechanism of action is comparable to the regularly used α-glucosidase inhibitors, such as acarbose, miglitol, and voglibose. Dietary therapy can incorporate anthocyanosides and other polyphenols into daily meals, relying on the natural slow-release effect of the food matrix and the synergistic effect of multiple components to achieve a more gentle and sustainable blood glucose regulation and to provide a sustainable and natural strategy for the prevention and long-term management of metabolic diseases.

### 3.3. Interaction of Phenolic Acid with Starch

Phenolic acid polyphenols are widely found in the plant kingdom, especially in tea, coffee and other common beverages, and their representative substances are caffeic acid, gallic acid (GA), cinnamic acid, etc. They can form hydrogen bonds with hydroxyl groups of starch molecules through phenolic hydroxyl groups and construct inclusion or non-inclusion complexes with the help of hydrophobic interactions or van der Waals’ forces; thus, they significantly enhance thermal stability and RS content while delaying blood glucose release to some extent to regulate blood glucose release. Crucially, the hydroxyl spatial arrangement defines their interaction modes: GA, with three adjacent hydroxyls, forms dense hydrogen-bonded networks within starch helices, elevating RS and thermal stability [22]; conversely, the extended conjugation of caffeic acid favors hydrophobic embedding but suffers steric hindrance in branched starch [31]; the single hydroxyl of cinnamic acid promotes amorphous complexes with dual α-amylase and dipeptidyl peptidase 4 (DPP4) inhibition [32]. These differences highlight how phenolic acid architectures lead to the tailoring of both the binding topology and physiological effects of the acids.

In a systematic study of the inhibition mechanism of DPP4, Li et al. pointed out that cinnamic acid exhibited optimal inhibitory activity with an IC50 value of 33.56 ± 1.13 mM and its binding process; DPP4 showed a spontaneous static burst mechanism and a mixed inhibition mode driven by hydrogen bonding as the main driving force, which revealed that cinnamic acid, while regulating the blood glucose response by complexing with starch, also has dual hypoglycemic potentials of prolonging the half-life of GLP-1 action and enhancing endogenous insulin secretion by inhibiting DPP4 [32]. In addition, caffeic acid and GA have become a hot research topic for their significant antioxidant properties and molecular stability.

Caffeic acid binds to the helical cavity of straight-chain starch through hydrogen bonding, strengthening the helical structure of the starch and making it difficult for the complex to be destroyed at high temperatures. It greatly increases the thermal stability of straight-chain starch [31]. Studies have shown that caffeic acid can increase the thermal decomposition temperature of high-temperature straight-chain corn starch, enhance its stability during processing and improve its antioxidant properties. Compared with caffeic acid, GA—which has a smaller molecular structure and more hydroxyl groups—can bind to straight-chain starch through the formation of strong hydrogen bonds embedded in the helical cavities of the starch and promote the stability of the complex. This binding not only increases the RS content of starch but also significantly slows down the digestion rate of starch and improves its antioxidant capacity, thus enhancing its health functions, especially in blood glucose regulation [24,32].

Furthermore, in addition to studying the mechanism of phenolic acid and starch complex formation, Li et al. investigated the inhibitory effect of cinnamic acid on DPP4 activity; they put forward the viewpoint that the adaptive inhibition of cinnamic acid on DPP4 activity occurs through the induction of spatial structural changes in the enzyme. This theory extended studies on the synergistic effect of polyphenol–starch to the maintenance of GLP-1 activity and thus blood glucose regulation through the inhibition of DPP4 activity [32].

### 3.4. Interaction of Lignan Polyphenols with Starch

Protocatechuic acid (PA) and soy isoflavones (SIs) are lignans, which are polyphenols with strong antioxidant properties that can be attributed to the abundance of phenolic hydroxyl groups and the extension of conjugated systems. PA has a small molecular structure with many hydroxyl groups, allowing it to form strong hydrogen bonding connections with hydroxyl groups in straight-chain starch molecules [23]. This bonding enhances the resistance of starch and reduces its digestion rate. Meanwhile, the antioxidant properties of PA help to improve the stability of the complex and delay the aging process of starch. SIs can enhance the RS content of starch by binding to branched-chain starch through hydrogen bonding. The binding of soy isoflavones to starch not only enhances the antioxidant properties of starch but also improve its digestive properties [33]. In addition, the isoflavone complexes contribute to the nutritional value and health functions of foods by altering the crystalline structure of starch.

In conclusion, polyphenols can be combined with starch through hydrophobic interactions, hydrogen bonding, electrostatic interactions and π–σ interactions to form a V-shaped inclusion and network cross-linking structure; this structure can significantly increase the content of RSs and slow down its digestion rate. Moreover, it has been found to enhance the thermal stability and antioxidant and anti-inflammatory activities of the complexes, effectively lower the glycemic index and improve the functional properties of the food. These characteristics are summarized in Table 1.

## 4. Effect of Starch–Polyphenol Complexes on Physiological Indices

The physiological role of starch in the human body can be understood in terms of evolutionary adaptations, digestive rates and metabolic effects. From an evolutionary perspective, the human ‘thrifty gene’ has led us—in times when energy is limited—to prioritize eating foods high in carbohydrates, so that we may quickly satisfy our physiological needs [1,2]. However, this genetic propensity has led to excessive carbohydrate consumption, which is a risk factor for metabolic illnesses such as obesity and type 2 diabetes [3,7]. Frugal genes make it easier for humans to obtain fast energy from RDSs but also make it difficult for the rapid blood glucose fluctuations associated with excessive RDS intake to be fully balanced by metabolism. Understanding the interaction between our genes and the environment can be helpful in precisely designing low-GI functional meals that are loaded with SDSs and RSs, or starches combined with polyphenols. Such a design could strike a balance between evolution-driven energy demands and modern dietary health management requirements in order to effectively prevent and treat metabolic disorders.

Polyphenols, as plant secondary metabolites, confer important protective functions to plants themselves and play multiple roles in human health and modern nutritional management. First, polyphenols in plants help to protect against ultraviolet radiation, pathogens and oxidative damage through their typical astringent and astringent properties; they are also involved in the formation of flower color and flavor [5]. For example, the astringent taste of tannins is a natural mechanism through which plants can attract or repel herbivores. Polyphenols’ hydroxyl structure can scavenge free radicals, reduce oxidative stress and regulate inflammatory pathways like NF-κB, preventing cardiovascular disease, cancer and other chronic diseases [8,35].

During blood glucose metabolism, polyphenols can form complexes with starch through hydrogen bonding, hydrophobic interactions and other noncovalent binding modes, interfering with the pasting and aging process of starch and specifically inhibiting the activities of α-amylase and α-glucosidase; this can significantly reduce the digestion rate of carbohydrates and postprandial blood glucose fluctuations, demonstrating a potential antidiabetic value [34,36]. Polyphenols’ digestive resistance property can delay carbohydrate digestion and inhibit α-amylase and α-glucosidase activity. The ‘thrifty gene’ turns the ‘anti-digestive’ properties of polyphenols into an opportunity for health interventions: through the polyphenol–starch complex, which slows down the absorption of carbohydrates while preserving the antioxidant and anti-inflammatory activities of polyphenols, a dual control of blood sugar and inflammation can be achieved. This allows for the dual regulation of blood sugar and inflammation [10,11]. Furthermore, polyphenols have anti-cancer potential through their inhibition of the cancer cell cycle and their activation of apoptosis signaling pathways [8,35].

Thus, we believe that polyphenols have a double significance: on the one hand, they are ‘defense factors’ for the survival and reproduction of plants; on the other hand, for human beings, the moderate use of their ‘anti-digestive’ properties and rich physiological activities can be transformed into a natural barrier for the maintenance of health and the prevention of chronic diseases in modern society, where there is, broadly, enough—or even too much—nutrition.

Starch–polyphenol complexes exhibit unique synergistic effects in physiological actions, which are particularly important in blood glucose management. Through intermolecular interactions, the complexes slow down the rate of enzymatic starch degradation, convert high-GI diets into low-GI ones and dramatically increase RS content compared to single components. The complexes have been shown to decrease GI in both in vitro and in vivo experiments; research on animals has shown that they produce smoother postprandial blood glucose profiles [11]. This ‘slow-release’ mechanism is not a unidirectional inhibition. The polyphenol–starch complex not only reduces the rate of carbohydrate digestion but also enhances antioxidant and anti-inflammatory activities by increasing the bioavailability and synergistic effects of the polyphenols themselves. This results in a more sustained effect against oxidative stress and inflammation. For instance, the HAPS-PLP complex has demonstrated optimal radical scavenging in antioxidant tests [10].

The ‘multi-mechanism synergy’ of starch–polyphenol complexes is in contrast with the ‘targeted efficiency’ of drugs. With an IC_50_ value of only 0.1 μM, acarbose directly blocks the interaction between the substrate and the catalytic center, reducing Vmax by 90% at the level of the conformational relationship through competitive bindings to the active site of α-amylase (K_m_ = 2.3 mM). This is far more effective than common natural polyphenols and medications [22]. Nevertheless, certain starch–polyphenol complexes, such as quercetin, are hydrogen-bonded to straight-chain starches and hydrophobically buried in the helical cavities of straight-chain starches. Starch–polyphenol complexes, like quercetin, exhibit multi-point contacts through hydrogen-bonded interactions to the surface of branched-chain starch and hydrophobic interactions embedded in the helical cavities of straight-chain starches. The synergistic effect of the hydrogen bonding network and V-type encapsulation exhibits multi-target and multi-mechanism synergism, having a lower inhibitory efficacy than acarbose [20]. With 50 mg of K per day, acarbose displays a linear dose–response curve at the quantitative relationship level. However, it was found to have a narrow safety threshold, with the incidence of abdominal distension increasing noticeably to 32% at doses greater than 100 mg per day. With an EC_50_ of roughly 5% *w*/*w*, the starch–polyphenol complexes displayed a typical S-type dose–response curve. The RS content increased steadily from 8.3% to 32.5% as the polyphenol concentration increased from low to high and the cell viability was consistently above 80%, indicating a wider safety window. Therefore, natural polyphenol-focused precision nutritional intervention strategies can effectively delay starch digestion and reduce GI while balancing multi-targeted physiological modulation with broader safety advantages, providing a feasible and low-risk natural solution for the long-term prevention and management of chronic metabolic diseases. Starch–polyphenol composites are now used in smart packaging, gut health and other areas beyond standard nutritional supplements. Pterostilbene polyphenols incorporated into starch–PVA films not only enhance the antioxidant properties of packaging but also realize pH-responsive phenol release, which can be used to monitor food freshness in real time [37]. Meanwhile, emerging research is exploring the effect of these complexes on the regulation of intestinal flora, with a view to providing new strategies for precision nutrition and chronic disease management through ‘nutrition–microecology–health’ interventions [38].

In general, starch–polyphenol complexes offer a novel concept for contemporary functional foods and accurate nutritional interventions through ‘dual regulation’, which not only reduces GI and slows down starch digestion but also increases the physiological activity of polyphenols and offers a natural remedy for metabolic disorders brought on by overnutrition. It also provides an effective natural solution to deal with metabolic diseases caused by overnutrition.

## 5. Characterization Techniques for Starch–Polyphenol Complexes

It is challenging to completely understand the multilayer features of starch–polyphenol complexes using a single analytical method when examining their structure–function relationship. Thus, the subsequent section methodically combines the techniques of structural analysis, compositional analysis, surface and morphological analysis, thermal stability analysis and phase transition analysis from various viewpoints, including molecular structure, surface morphology, thermal behavior and chemical composition. The methods are complementary to each other, from the macro- to the microscopic scale, from the static to the dynamic in form and encompass a range of activities from physicochemical to biological; accordingly, they build a technical framework for the characterization of complexes in an all-round way, which provides solid technical support for the optimization of the preparation process, the enhancement of functional properties and the development of functional foods.

### 5.1. Digestive Characterization

Digestive properties are important characteristics of starch as a food resource. Englyst et al. performed in vitro enzymatic digestion of starch samples with pancreatin and amyloglucosidase at 37 °C under physiological conditions. They terminated the reactions at 20 and 120 min, respectively, and then quantified the amount of blood glucose released in the reaction solution using the glucose oxidase–peroxidase (GOPOD) method. After the reactions were terminated at 20 min and 120 min, the amount of blood glucose released from the reaction solutions was quantified using the GOPOD method. Accordingly, the starches were categorized as RDS, SDS or RS according to the enzymatic rate at different time points. This was performed with the aim of supporting the concept that RSs can escape from the small intestinal enzymatic degradation to enter into the colonic fermentation, which was proposed in order to evaluate the physiological function. Singleton et al. further constructed a three-stage in vitro co-digestion model including oral salivary amylase, pepsin and the acidic environment, using small intestinal pancreatic amylase and bile salts; they continuously monitored the sugar release using the 3,5-dinitrosalicylic acid (DNS) method or the GOPOD method, respectively. This was performed at each stage in order to mimic human digestive kinetics and to assess the changes in the performance of the starches. The starches were mechanically ball-milled and polyphenol-coated. The changes in the properties of the starches were modified by the mechanical ball-milling process with polyphenol encapsulation occurring during the enzymatic digestion process. According to the results, the initial ball-milling treatment led to a temporary decrease in digestibility due to the physically disrupted starch crystalline regions. However, the polyphenol molecules created strong hydrogen bonds between the starch chains by overlapping the phenolic hydroxyl groups with the σ–σ* orbitals of the starch hydroxyl groups. This was made possible through the hydrophobic interactions between the aromatic ring π–electrons and the helical cavity C–H orbitals. The interaction between the π–electron of the aromatic ring and the C–H orbital of the helix cavity creates a molecular barrier between the starch chains, which is difficult to recognize using digestive enzymes. This finding significantly slows down the rate of enzymatic degradation and dramatically decreases the release of blood glucose in the three-phase digestive system; thus, direct physiological evidence for the development of low-GI functional foods by fortification was obtained [39].

### 5.2. Compositional and Structural Analysis

Composition and structure correlate to the properties of the substance itself, which then determine the action of a substance. The researchers used a multi-technique combination of traditional instrumental analyses to study and characterize starch, polyphenols and their complexes. Galani used a strategy combining gel permeation chromatography (GPC) and liquid chromatography–electrospray ionization tandem mass spectrometry (LC-ESI-MS/MS) to accurately quantify the content and molecular weight distribution of the components in the polyphenol–amylopectin complexes; additionally, they assessed the correlation between the complexation efficiency and functional properties. PC relies on different pore size fillers for the hierarchical interception of molecular chain length, which can visually reflect the migration of the molecular weight of starch from the high-polymerization interval to the middle- and low-polymerization intervals after compounding; in contrast, LC-ESI-MS/MS uses chromatographic separation together with a multi-reaction monitoring mode to achieve the sensitive identification and quantification of polyphenol compounds at the microgram level. LC-ESI-MS/MS uses chromatographic separation together with the multi-reaction monitoring mode, which can realize the sensitive identification and quantification of polyphenol compounds on the microgram level. The experimental results showed that, following starch–polyphenol complexation, the molecular weight distribution of starch shifted significantly to the low-molecular-weight direction; moreover, the amount of polyphenol had a significant positive correlation with both its own molecular weight and its antioxidant activity. This finding not only provides a reliable basis for the quantification of polyphenol adsorption and starch chain length distribution, but also lays a solid foundation for the revelation of complex binding efficiency and strength of action [40].

The structural transformation brought about by noncovalent interactions and the complexation of polyphenols and amylopectin was revealed in five dimensions using the following five different methods: FTIR, XRD, small-angle X-ray scattering (SAXS), nuclear magnetic resonance (NMR) and the Standard Test Method for Tensile Properties of Thin Plastic Sheeting (ASTM D-882 tensile test). These five techniques were used to systematically demonstrate the structural change in polyphenol–starch caused by complexation and noncovalent interactions: molecular vibration, crystal conformation, microstructure, chemical shift and macroscopic mechanical properties [5,41]. Using Cu Kα rays (λ = 0.1542 nm), Nara and Komiya employed XRD to gather diffraction patterns. These revealed that the initial A-type or B-type diffraction peaks were greatly diminished or vanished, along with the appearance of a peak in the V-type inclusion complexes and a general decline in the degree of crystallinity [42]. SAXS polyphenol–starch complexes can be used to characterize the microstructure of the complexes on the nanoscale by measuring the scattering intensity of incident X-rays; this is possible due to the differences in electron densities in the samples. It is thus possible to quantitatively obtain information on the periodic and disordered structure of the system, which shows that polyphenol doping could significantly inhibit the formation of the original periodic nanostructures of starch [43]. The quantitative results showed the changes that occurred in the peak widths of the solid-phase 13C CP/MAS NMR and liquid-phase 1H NMR. These were highlighted through chemical shifts, confirming the existence of hydrogen bonding and hydrophobic interactions between polyphenol hydroxyl groups and starch chains [44]. Additionally, the tensile test—conducted in accordance with the ASTM D-882 standard—revealed that complexes with low polyphenol content could have significantly better mechanical properties, while complexes with higher polyphenol content showed a decrease in strength and ductility. These findings demonstrated the impact of polyphenol–starch interactions on the macroscopic mechanical properties of the dose-dependent effect [45]. The above multi-scale and multi-method data synergistically demonstrated the correlation between the microscopic binding mechanisms and the macroscopic properties during the compounding process; this provided a basis for an in-depth understanding to be built of the structural stability of the complexes as well as the optimization of functional product design.

### 5.3. Thermodynamic Behavior and Stability Analysis

A thorough investigation of the thermodynamic behavior of starch–polyphenol complexes can reliably define their phase transition temperatures and exothermic (heat absorption) properties. Such an investigation can also show how the materials decompose over time during high-temperature processing and storage, which can serve as a foundation for the optimization of functional food processes. Carter et al. used differential scanning calorimetry (DSC) experiments to accurately record the heat absorption and exothermic peaks of the samples by gradually increasing the temperature. They discovered that the polyphenol molecules’ embedding in the helical cavities of the starches significantly decreased the onset temperature of pasting and promoted the transition of the starches from a crystalline state to an amorphous state. The V-type inclusion displayed a notable heat absorption peak at roughly 130 °C, indicating the complexes’ changed thermal behavior [46]. Deng et al. employed thermogravimetric analysis (TGA) to track the mass loss curve over the entire process, clearly separating the pyrolysis portion of the starch skeleton in the 250–300 °C region from the water volatilization stage, which was below 150 °C. The results showed that, although the overall pyrolysis temperature decreased, the complexes showed enhanced high-temperature tolerance due to the reorganization of the internal structure of the granules. Additionally, Zhang et al. discovered that, in the TGA of the caffeic acid–corn starch system, the pyrolysis temperature of waxy corn starch composite samples slightly increased, while that of normal- and high-molecular-weight straight-chain corn starch composites correspondingly decreased. This phenomenon is a direct reflection of the differential effects of polyphenol molecular weight and hydroxyl group distribution on the thermal stability of the complexes [31,47].

Brodkorb employed the ITC technique to examine the stability of the starch–polyphenol complex system. The findings demonstrated that the binding constants of polyphenol along with starch ranged from 1.25 × 10^4^ to 5.14 × 10^4^ m^−1^ and the entire binding process was an exothermic reaction (ΔH < 0), demonstrating the thermodynamic driving force of spontaneous binding as well as the two molecules’ complete affinity. In addition to the optimal ratio of polyphenols to starch, these quantitative thermodynamic data provide a theoretical basis for creating a deeper understanding of the binding mechanism and the formulation design of functional foods [48].

### 5.4. Molecular Docking and Molecular Dynamics Simulations

**Figure 4:**
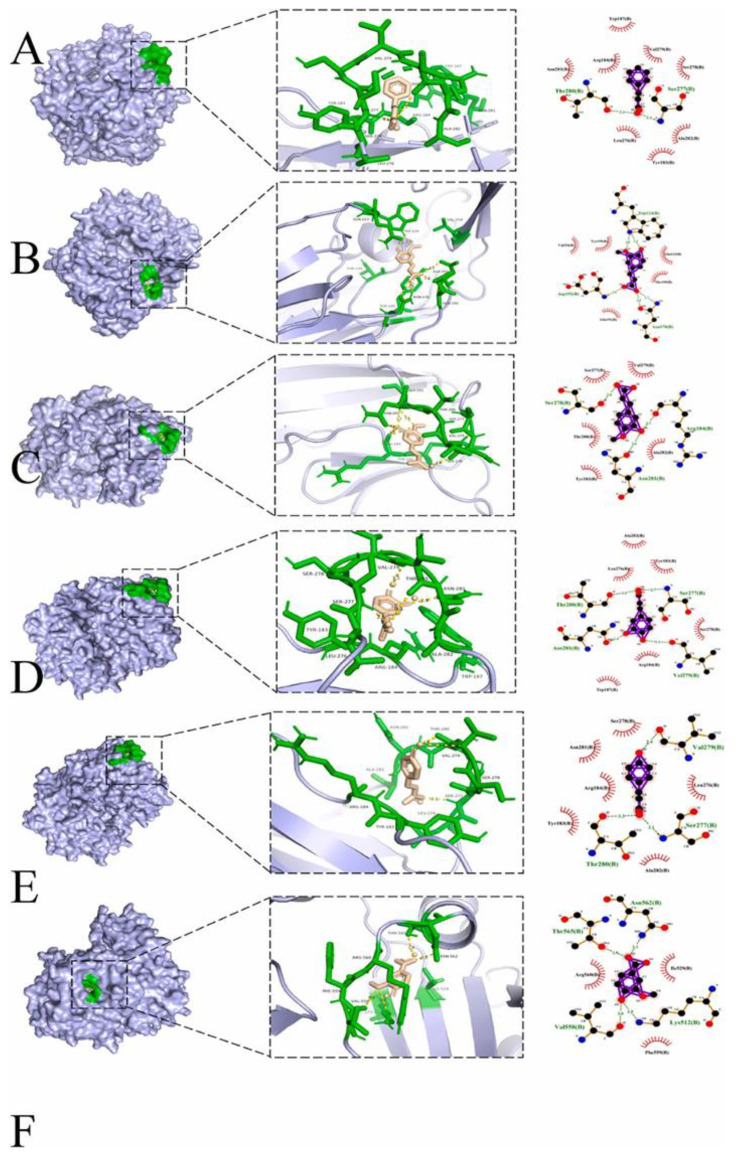
Molecular docking analysis of cinnamic acid (CIA) and its derivatives with DPP4. (**A**–**F**) Three-dimensional (**left**) and two-dimensional (**right**) binding modes of CIA (**A**), ferulic acid (**B**), isoferulic acid (**C**), caffeic acid (**D**), protocatechuic acid (**E**) and vanillic acid (**F**) within the active site of DPP4. (**F**) Acid within the active site of DPP4. Adapted with permission from the authors of Ref. [32], 2024: Jiaying Li, Xiaoping Yang, Chunhong Xiong, Jinsheng Zhang and Ganhui Huang.

The purpose of molecular docking and molecular dynamics simulations was to use computer modeling to evaluate the binding locations and binding energies of starch and polyphenols. Using programs like AutoDock, GROMACS and Gaussian, the purpose is to construct intricate models of starch (straight and branched chains) and polyphenols (EGCG, quercetin, etc.) and to quantitatively describe their intermolecular interactions by computing parameters like the number of hydrogen bonds and the binding free energy. This innovative technology method is a major component of current research on the openness and ease of visualization. Bannwarth et al. combined the structural models of amylo-oligosaccharides available from the CAZy database (CAZy-Home) with the structural models of polyphenols (such as quercetin, epigallocatechin gallate or EGCG) downloaded from the PubChem database. This was performed using the GFN2-xTB method, which was implemented in the open-source xtb quantum chemistry software and its aISS (Automated Interaction Site Screening) module for automated interaction site screening with the CREST program. The CAZy database (CAZy-Home) provides structural models (straight and branched chains) and the 3D coordinates of polyphenol molecules (e.g., quercetin and epigallocatechin gallate, or EGCG) that can be downloaded from the PubChem database for efficient molecular docking studies of starch and polyphenol molecules. The simulation results indicated that the free energy of binding of short-chain starch to EGCG was −26.73 kcal/mol, while that of long-chain starch was −23.20 kcal/mol, which indicated that the short-chain system was more tightly bound to the polyphenols. Additionally, it was established that the polyphenols’ B-ring serves as the primary binding site. We utilized the ‘3D + 2D’ visualization approach developed by Li et al. in the docking research of DPP4 with phenolic acid derivatives to the starch–polyphenol system to make these static docking results easier to read and understand. Li et al. presented results for a three-dimensional visualization framework of starch–polyphenol complex docking results, which were compared with those of the two-dimensional framework—see Figure 4. In the 3D view, a representative polyphenol molecule is caught inside the starch single-helix cavity. Its hydrophobic aromatic ring forms a continuous channel with the hydrophobic surface of the helix exterior; the dashed lines show the hydrogen bonding interaction between the phenolic hydroxyl group and the hydroxyl group of the nearby sugar residue. The main hydrophobic contact regions are accurately indicated by fan arrows in the 2D network diagram; the notes provide a list of the types and distances of each individual hydrogen bonding and van der Waals interaction, providing a quick overview of the binding conformation and local interactions [32]. Based on this static visualization, Li’s group then ran 100 ns molecular dynamics simulations of the ideal docking conformation to confirm the accuracy of the static docking prediction and demonstrate how hydrophobic channels and hydrogen bonds work in concert to keep the system stable. These thermodynamic and kinetic molecular data not only provide strong evidence for the elucidation of the composite mechanism, but also lay a theoretical foundation for subsequent design work in structural modification and functional optimization [11,49].

Molecular docking software with similar functions include AutoDock Vina [50], GOLD [51], LeDock [52], Glide [53], ClusPro [54], Hex [55] and SwissDock [56]. Molecular dynamics simulation software include GROMACS [57], AMBER, CHARMM [58], NAMD [59], LAMMPS [60], DESMOND [61] and GROMOS [62]. These are widely used in the fields of structural biology, drug design and material simulation.

### 5.5. Particle Characterization and Rheological Analysis

Chen et al. conducted a thorough analysis using Herschel–Bulkley model fitting (HBMF), the Steady-State Shear and Dynamic Oscillatory Rheology (SOSDOR) tests and Dynamic Light Scattering (DLS) to assess the dispersion stability and processing performance of the complexes in the aqueous phase and to provide a physical basis for the optimization of high shear process parameters such as extrusion and 3D printing. The DLS was performed by determining the particle size distribution through the Brownian motion diffusion coefficient (BMDC). DLS reduces the particle size distributions and calculates the Brownian motion diffusion coefficient of the particles to reveal the bimodal structure of macroscopic aggregates (189–340 nm) and nanoscale (8–23 nm) complexes. The rheological test determines the variation in the energy storage modulus, G′, the loss modulus, G″, and the loss factor, tanδ, with the frequency. The results show that G′ ≫ G″ and tanδ < 1; the elastic response is enhanced with the increase in temperature. The Herschel–Bulkley model fitting approach indicates typical pseudoplasticity by showing that the yield stress, σ_0_, and viscosity coefficient, K, increase significantly with temperature and that the flow behavior exponent, n, is always less than 1 and decreases further. It also offers important references for the industrial processing of the complex and for optimizing the processability of derived products [63,64,65].

### 5.6. Functional Activity Analysis

A comprehensive assessment of the physiological functions of starch–polyphenol complexes can be performed using fluorescence/free radical scavenging assays and cellular assays. Fluorescence/radical scavenging assays directly reflect the antioxidant activity of the complexes by determining their scavenging rate or reduction capacity for specific free radicals. This study shows that the antioxidant properties of polyphenol–starch complexes are not only fully preserved but are even enhanced in some systems, which provides strong support for the development of highly functional foods [66]. The results showed that the antioxidant properties of polyphenol–starch complexes were not only fully retained but even enhanced in some systems, which provided strong support for the development of highly functional foods. A cellular assay was used to examine the complex’s impact on the in vitro proliferation of cultured cells. The findings indicated that the cells’ survival rate was over 80% at the treated concentrations, confirming the complex’s good biocompatibility and laying the groundwork for future in vivo safety evaluations [67].

### 5.7. Other Detection Methods

A number of ‘other assays’—including thin-layer chromatography (TLC), iodine binding assays and the DMSO solubilization () method—have been used in addition to traditional characterization techniques to study starch–polyphenol complexes. These assays are used to supplement the multidimensional analysis of mechanical properties, compositional optimization, macroscopic–microscopic structures and functional activity. Since non-targeted metabolomics and chemometrics are incorporated into big data statistics ideas, they have become more significant. Phenolic components in starch–polyphenol complexes and their bio-accessible binding have been thoroughly identified through chemometrics and non-targeted metabolomics techniques. First, the metabolite data of the complexes were collected using ultra-high-performance liquid chromatography with full spectrum time-of-flight mass spectrometry (UHPLC-QTOF-MS/MS). Chemometrics methods such as principal component analysis (PCA) were then used to extract the primary difference molecules from the huge dataset. The findings revealed distinctive polyphenol molecules like protocatechuic acid and quercetin as biomarkers that can be used to both quantitatively evaluate the complexes’ bioaccessibility and uncover their possible health benefits. These findings indicate that the complexes provide a strong molecular foundation for future precision nutritional interventions and the creation of functional foods [68].

In conclusion, this work thoroughly examines the multilevel properties of starch–polyphenol complexes, ranging from molecular binding to functional expression; moreover, the researchers have established a comprehensive detection system that reveals the complexes’ binding mechanism, thermal stability, anti-enzymatic properties and bioactivities in a synergistic manner—ranging from molecular vibration and crystalline transformation to microscopic morphology, thermodynamic parameters and physiological functions. This lays a strong basis for the accurate design and optimization of low-GI functional foods. In this review, the technical categories, principles and accessible core information are summarized in Table 2, while the comparative advantages and disadvantages of each characterization technique are outlined in Table 3.

## 6. Practical Applications of the Developed Starch–Polyphenol Complexes and Products

Research on foods with low GI and low GL has increased due to the growing demand for healthier diets. Starch–polyphenol complexes have been used gradually in a variety of food formulations because of their unique advantages, which include glycemic response management, enhanced food functionality, RS as a carbon source for gut microorganisms and polyphenols for intestinal microbiota screening. A detailed discussion on the use of starch–polyphenol complexes in real food products and the creation of their derivatives is presented in the following subsections.

### 6.1. Development of Low-GI Foods

In current food research and development, the production of low-GI meals has been a focus of research in response to the problem of rapid postprandial blood glucose spike linked with high-GI (glycemic index) foods. It is possible to create resistant molecular structures that are more difficult for enzymes to recognize by combining polyphenols with starch to form complexes. This can significantly increase the amount of RSs present and slow down the rate at which starch is digested, thereby lowering the GI. For instance, in an in vivo experiment, complexing green tea polyphenols with rice starch lowered the peak of postprandial blood glucose and significantly increased the amount of RS [9]. Low-GI meals are now nutritionally controlled and are health-promoting due to the addition of natural polyphenols such as anthocyanins and GA, which further improve their antioxidant and antibacterial properties. Among them, the design of low-GI healthy snacks has also benefited from the starch–polyphenol complex strategy. By utilizing the antioxidant and delayed digestion qualities of tea polyphenols, adding them to potato chips or corn crackers not only dramatically lowers their GI values but also improves the health benefits of the snacks. Through enhancing fullness and regulating caloric intake, these creative snacks offer a workable way to manage weight and treat metabolic disorders brought on by overeating [9]. Starch–polyphenol composite technologies are setting the standard for more accurate, sustainable and healthful functional foods, especially when combined with the general development of low-GI foods.

### 6.2. Functional Bakery Products

Making useful baked goods is another popular use for starch–polyphenol complexes. Bakery products, such as bread and cookies, are often high in GI refined starch, which can easily contribute to a quick rise in blood glucose levels following meals. Starch–polyphenol complexes can effectively lower a food’s GI rating, making it appropriate for those with diabetes or those who must regulate their blood sugar levels. For instance, research has demonstrated that adding tea polyphenols or baobab fruit extract to bread can considerably lower the postprandial glycemic response [11]. In addition, starch–polyphenol complexes can be used to develop high-fiber cookies, replacing some of the flour and increasing the dietary fiber content of the food [70].

### 6.3. Functional Drinks

Beverages, as part of a daily diet, are sometimes disregarded for their effect on blood glucose levels and weight management. As the demand for healthy beverages grows, the use of starch–polyphenol complexes in the beverage industry is gradually rising, particularly in the production of low-sugar and functional drinks. By combining natural polyphenols, such as those found in tea, with anthocyanins, drinks can not only offer health benefits—including anti-inflammatory and antioxidant properties—but they also successfully reduce the absorption of sugar as well as preserve a steadier blood glucose response. For example, PRRBAE can considerably lower the activity of starch-digesting enzymes, decreasing the rate of starch digestion in the drink and generating a sugar-control effect [15]. In this way, the beverage lessens the possible harmful health effects of overeating in addition to assisting with blood glucose regulation.

### 6.4. Functional Starch-Based Films

Starch–polyphenol complexes are also widely used in the development of food packaging materials, especially in the field of antioxidant packaging and smart packaging. Starch-based films are an ideal food packaging material due to their natural origins and degradability. The addition of polyphenols enhances the antioxidant capacity of the film and extends the shelf life of the food. For instance, a starch–PVA composite film with butterfly pea flower extract was shown to successfully prevent food from oxidizing, without compromising the film’s mechanical qualities [37]. In addition, starch–polyphenol composite films can release polyphenols in response to environmental pH changes, allowing real-time monitoring of food freshness [71].

### 6.5. Medicinal Diets and Foods for Special Medical Purposes

Starch–polyphenol complexes have shown significant advantages in the field of formulas for special medical purposes (FSMPs). First, by postponing the digestion of starches and promoting fullness, its low GI properties and high RS content not only help diabetic patients maintain stable blood glucose levels but also offer a safe and efficient dietary intervention for weight reduction and the requirements of older adults [72]. For example, because of their poor digestion, gorgonzola starch–polyphenol complexes have been proposed as a staple meal substitute for diabetic people. The high RS content of these foods can reduce calorie intake and contribute to weight management [69].

However, despite their greater efficacy and selectivity and their potential to effectively address diseased processes in the near term, traditional medicines frequently have noticeably adverse physiological effects. Meanwhile, natural product foods—such as dietary supplements containing starch–polyphenol composites—are recognized for their mild and long-lasting modulation properties and are less likely to cause negative side effects. Polyphenols can enhance general health and fend off chronic illnesses by slowing down the rate at which starch is digested, working in concert with their antioxidant and anti-inflammatory properties [8,35].

The lack of fully developed national norms and regulations has hindered the widespread use of starch–polyphenol composite meals for special medicinal purposes, despite their dual benefits in terms of functionality and safety. However, in the field of scientific research—specifically, medicinal dietary research—interest in the starch–polyphenol interaction mechanism is increasing; combined with the latest advances in molecular biology and genomics, it is expected that development in this area will lead to designs that can meet individualized nutritional needs, with precise intervention effects for functional FSMPs. Accordingly, research in this area will provide a new way of thinking in relation to the prevention and treatment of metabolic diseases, such as diabetes and obesity [11,38].

### 6.6. Three-Dimensionally Printed Foods

With the development of food science and technology, 3D printing technology has been introduced into food processing. Starch–polyphenol complexes have become an important raw material for low-digestibility 3D-printed foods due to their good rheological properties and printability. For example, a starch complex with 5% GA added shows an increase in RS content from 22.10% to 53.36% while maintaining excellent printability [73]. In addition to satisfying individual nutritional needs, this low-GI 3D-printed food can be customized in terms of appearance and taste.

### 6.7. Commercialized Products and Market Prospects

Currently, although starch–polyphenol complexes have made significant progress in laboratory studies, most of the products are still in the research and development stage and have not been widely commercialized. Most of the low-GI foods and functional foods mentioned in the article are in the laboratory research stage or the small-scale production stage, and have not yet become mainstream market products. The lack of fully developed national norms and regulations has prevented the widespread use of starch–polyphenol composite meals. Scaling up to commercial production also entails higher costs for sourcing and purifying polyphenols, adapting existing equipment, and implementing process controls; moreover, technological hurdles are faced in attempts to achieve consistent complex formation, maintain stability under high-temperature processing and ensure sufficient shelf-life. Overcoming these regulatory, economic and technological barriers will be critical to unlocking the market potential of starch–polyphenol complexes. Nonetheless, starch–polyphenol complexes are anticipated to play a significant role in the future of the health food market due to the growing demand for functional foods and increasing consumer health consciousness, particularly in the treatment of diabetes and diets for weight loss, along the nutritional requirements of the aging population.

## 7. Conclusions and Outlook

Starch–polyphenol interactions significantly alter the physicochemical properties and digestive behaviors of starch through noncovalent forces such as hydrogen bonding, hydrophobic interactions and electrostatic binding. By inhibiting α-amylase activity, polyphenols are inserted in the spiral cavity or surface of starch to form a V-type inclusion complex, which not only raises RS content but also diminishes the postprandial blood glucose peak and slows the rate of digestion. This ‘dual regulation’ process offers a natural approach for dietary interventions in metabolic illnesses by postponing the release of blood glucose while boosting the antioxidant and anti-inflammatory properties of polyphenols.

Although starch–polyphenol complexes show great promise in regulating starch digestibility and developing functional foods, there are still many unanswered questions in current research. Unlike drugs that have precisely targeted objects of action with clear and efficient effects, natural products in food interact weakly and in a reversible manner; in addition, they require enhanced synergies between the various food components to achieve their effects. Among these, the most notable is the dearth of systematic research on evaluating the complexes’ bioavailability during in vivo metabolism and their long-term health impacts, which limits their legitimacy and the promotion of their application in precision nutrition interventions. Meanwhile, the complex three-dimensional binding mode between polyphenols and starch and its structure–function correlation have not been fully elucidated, meaning that the mechanism of the complex system remains unclear. Furthermore, the existing literature focuses primarily on the interaction of single polyphenols with specific starch fractions, lacking systematic comparative studies on the structure of multiple polyphenols with different starch types; this means that it is difficult to provide a comprehensive guide for compounding law and application strategy.

To better understand the role of food therapy, future research should combine molecular dynamics simulation and in vivo experimental methods to build a theoretical and technical system. For example, it should systematically explore the dynamic binding mechanism of the starch–polyphenol complex and its metabolic pathway in the digestive process. Future research should also strengthen multidisciplinary integration, track the therapeutic mechanism of drugs and partially follow the evaluation method of drugs, in addition to reflecting the high content and variety of food-borne natural products. For instance, we can systematically explore the dynamic binding mechanism of starch–polyphenol complexes and their metabolic pathways in the digestive process to deepen our understanding of their functionality. Simultaneously, we can precisely demonstrate the relationship between the complexes’ conformational changes and physiological functions with the aid of high-throughput technologies such as SAXS and non-targeted metabolomics. We can also offer data support for the development of an integrated structure–performance–application model. On this basis, by optimizing the compounding conditions and processing techniques, the process adaptability and nutritional stability of the products can be improved, helping to promote their commercialization in the direction of low-GI food and smart packaging materials. With the deepening of theoretical research and application development, starch–polyphenol complexes are expected to be more essential in precise nutritional intervention, chronic disease management and the construction of sustainable food systems. Finally, inspired by the pipeline used in pharmaceutical development, future work should establish a closed-loop process that begins with in vitro screening, moves through detailed structural characterization and in vivo validation, continues with process optimization and ends with comprehensive functional evaluation; thus, a robust structure–performance–application framework can be formed which can accelerate the real-world deployment of low-glycemic-index foods and intelligent packaging solutions.

## Figures and Tables

**Table 1:** Comparative analysis of interaction differences between various polyphenol classes and starch types.

Polyphenol Class	Major Representative	Binding Mode	RS Increase (%)	Δ Thermal Stability (°C)	Reference
Flavonoids	Qu	Hydrophobic interaction and hydrogen bonding	15–25	+25	[9]
Phenolic acids	CA	Hydrogen bonding (amylose helix inclusion)	10–18	N/A	[31]
Anthocyanins	Purple rice bran anthocyanin extract (PRRBAE)	π–σ interactions and hydrogen bonding	20–30	N/A	[34]
Lignans	PA	Strong hydrogen bonding	12–22	N/A	[23]

**Table 2:** Characterization techniques for starch–polyphenol complexes.

Category	Technique/Method	Principle	Analytical Content	Key Information	Reference
Component analysis	GPC	Separation by chain length	Component content	Polyphenol adsorption amount	[3]
	LC-ESI-MS/MS	Quantitative MS	Molecular weight distribution	Starch chain length distribution	
Structural analysis	FTIR/ATR-FTIR	Molecular vibration	Chemical bonds	OH-stretching peak shifts	[9]
	XRD	N/A	Crystalline forms	V-type characteristic peaks	[5]
	NMR	N/A	Binding sites	Chemical shift variations	[10]
Surface and morphology	SEM	Electron imaging	Particle surface morphology	Surface roughness; starch dispersion	[11]
	TEM	Transmission imaging	Internal nanostructure	Nanoscale morphology	[64]
	AFM	AFM scanning	N/A	Nanostructural topography	
Thermal behavior	DSC	N/A	Phase transition temperatures	V-type inclusion endothermic peaks	[69]
	TGA		Thermal decomposition	Decomposition temperature ranges	
Particles and rheology	DLS		Particle size distribution	Bimodal size distribution; pseudoplastic behavior	[23]
	Steady shear/dynamic oscillation tests	Rheological testing	Viscoelastic parameters	G′, G″, tan δ, σ₀, K and n	[9]
Digestive properties	In vitro digestion simulation	Enzymatic hydrolysis kinetics simulation	Glucose release curves at different stages	RDS/SDS/RS content; GI evaluation	[57]
Thermodynamics and modeling	ITC	Isothermal titration	Binding constants	ΔH, ΔS and ΔG	[34]
	Molecular docking/molecular dynamics	Computational modeling	Thermodynamic parameters	Binding modes and energies	[9]
	SAXS	N/A	Nanostructure	Microstructural order/disorder	
Functional activity	DPPH	Radical scavenging	Antioxidant capacity	Free-radical scavenging rates	[67]
	ABTS			Hydrophilic and lipophilic components	[38]
	FRAP	Ferric ion reduction to ferrous ion	Reducing power	Antioxidant potential	
	Cell assays	Cell proliferation assays	Biocompatibility	Cell viability	
Other methods	ASTM D-882 tensile	Mechanical testing	Mechanical properties	Tensile strength	[64]
	Untargeted metabolomics	MS-based metabolomics	Metabolite fingerprinting	Bioavailability biomarkers; polyphenol	[9]
	TLC	Chromatography	Separation efficiency	Semi-quantitation	
	Iodine binding	Colorimetry	Helical interference	Interference index	

**Table 3:** Comparative analysis of characterization techniques for starch–polyphenol complexes.

Technique	Advantages	Disadvantages	References
FTIR	Rapid, non-destructive; highlights specific bond shifts	Limited spatial resolution; overlapping peaks can complicate analysis	[5,9]
XRD	Definitive identification of crystalline forms and V-type peaks	Requires well-crystallized samples; bulk technique	[42]
NMR	Detailed molecular-level binding information; quantitative	Low sensitivity; expensive instrumentation; long acquisition times	[44]
SEM/TEM/AFM	High-resolution imaging of surface and nano-morphology	Sample preparation artifacts; only surface/topography information	[11,64]
DSC/TGA	Accurate phase-transition and thermal-stability data	No structural detail; can alter sample during heating	[46,47]
ITC	Direct measurement of binding thermodynamics	Large sample requirement; limited to soluble systems	[48]
Molecular docking and MD	Rich visualization of binding sites; predicts binding energies	Computationally intensive; may not capture full in vivo environment	[11,49]
DLS and rheology	Quantifies particle size distribution and flow properties in solution	Sensitive to polydispersity; requires well-dispersed suspensions	[63,64,65]
In vitro digestion	Simulates physiological starch breakdown; yields RDS/SDS/RS fractions	Simplified model may not reflect in vivo complexity	[39]

## Data Availability

No new data were created or analyzed in this study. Data sharing is not applicable to this article.
